# Expression Profiling of Long Noncoding RNA and Messenger RNA in a Cecal Ligation and Puncture-Induced Colon Injury Mouse Model

**DOI:** 10.1155/2020/8925973

**Published:** 2020-11-03

**Authors:** Jinxiang Huang, Yuan Liu, Qingqiang Xie, Guorui Liang, Haifan Kong, Meiling Liu, Yujie Wang, Shanshan Zhang, Xuefeng Li

**Affiliations:** ^1^The Sixth Affiliated Hospital of Guangzhou Medical University, Qingyuan People's Hospital, The State Key Laboratory of Respiratory Disease, Sino-French Hoffmann Institute, School of Basic Medical Sciences, Guangzhou Medical University, Guangzhou 511436, China; ^2^Shenzhen Luohu People's Hospital, The Third Affiliated Hospital of Shenzhen University, Shenzhen 518001, China; ^3^Nan Shan School, Guangzhou Medical University, Guangzhou 511436, China; ^4^Department of Gastroenterology, Affiliated Yantai Yuhuangding, Hospital of Qingdao University, Yantai 264000, China; ^5^Key Laboratory of Regenerative Biology, Guangdong Provincial Key Laboratory of Stem Cell and Regenerative Medicine, South China Institute for Stem Cell Biology and Regenerative Medicine, Guangzhou Institutes of Biomedicine and Health, Chinese Academy of Sciences, Guangzhou 510530, China

## Abstract

**Background:**

Emerging evidence reveals that long noncoding RNAs (lncRNAs) play important roles in the pathogenesis of sepsis. However, the detailed regulatory mechanisms of lncRNAs or whether certain lncRNA could serve as a biomarker in the septic colon remains unclear. The aim of this study was to investigate the profiles of lncRNAs and mRNAs in the septic colon through whole-transcriptome RNA sequencing and to reveal the associated regulatory mechanism.

**Method and Result:**

We established a mouse model of sepsis by cecal ligation and puncture (CLP). Colon samples were collected upon CLP or sham surgery after 24 h. Whole-transcriptome RNA sequencing was performed to profile the relative expressions of lncRNAs and mRNAs. 808 lncRNAs and 1509 mRNAs were differentially found in the septic group compared with the sham group. Bioinformatics analysis including Gene Ontology (GO) analysis and Kyoto Encyclopedia of Genes and Genomes pathway analysis (KEGG) was performed to predict the potential functions of these RNAs. GO analysis showed that the altered lncRNAs were enriched and involved in multiple immune responses, which may be a response to sepsis stress. KEGG analysis indicated that upregulated lncRNAs were significantly enriched in the p53 signaling pathway, NF-*κ*B signaling pathway, and HIF-1 signaling pathway. Downregulated lncRNAs were mostly found to be involved in tight junction, leukocyte transendothelial migration, and HIF-1 signaling pathway.

**Conclusion:**

Our results indicate that these altered lncRNAs and mRNAs may have crucial roles in the pathogenesis of sepsis. This study could contribute to extending the understanding of the function of lncRNAs in sepsis, which may help in searching for new diagnostic biomarkers and therapeutic targets to treat sepsis.

## 1. Introduction

Sepsis is defined as a syndrome of morbid multiorgan dysfunction caused by infection [1]. A series of complex pathological processes such as endothelial cell injury, cytokine and inflammatory factor release, and multiorgan failure are experienced upon sepsis [2], resulting in a mortality rate as high as 40% in intensive care patients [3]. Thus, the complexity of the etiology and pathogenesis of sepsis augments the challenge of the diagnosis and treatment of sepsis [4].

An injured intestine can aggravate the multiple-organ dysfunction, which is one of the leading causes of death in septic patients [5, 6]. As one of the important components of the immune system and the repository of intestinal microbiota, the colon plays a significant role in maintaining host homeostasis [7]. Gut integrity is compromised in abdominal sepsis with increase in cellular apoptosis and barrier permeability, which contributes to bacterial translocation and reduction of microbial diversity, propagating local damage and distant organ failure [8–11]. Recovering homeostasis of the intestinal environment may be an important candidate strategy for the treatment of sepsis [12, 13]. However, the molecular mechanism of sepsis-induced gut dysfunction remains to be further explored.

lncRNAs were defined as a large class of noncoding RNAs that are greater than 200 nucleotides, which represent a versatile class of molecules [14]. lncRNAs could affect many biological processes, such as cell proliferation, differentiation, and apoptosis at transcriptional, posttranscriptional, and epigenetic levels [15]. Therefore, lncRNAs could be involved in various human diseases [16–18]. Recently, sepsis-related lncRNAs have attracted scientists' attention. lncRNA profiles of a certain organ commonly associated with depression in sepsis have been documented [19, 20]. These studies implied that differential expression of lncRNAs is related to organ damage induced by sepsis.

Here, we want to explore whether lncRNAs play roles in sepsis-induced colon depression. To elucidate the features of lncRNAs in sepsis-induced colon depression, we constructed a CLP sepsis mouse model and collected septic colon tissues for whole-transcriptome RNA sequencing 24 h later. Bioinformatics analysis was performed to predict the biological functions and key signaling pathways of these differentially expressed lncRNAs. Moreover, we focused on one particular lncRNA (LINC233) for further research to determine the potential immune regulation mechanism. To this end, the purpose of this study was to explore the correlation between intestinal dysfunction caused by sepsis and underlying lncRNAs, to provide new sight for future study, and to find new diagnostic biomarkers and therapeutic targets for sepsis.

## 2. Materials and Methods

### 2.1. Mouse Model of Cecal Ligation and Puncture

C57BL/6J mice aged between 6 and 8 weeks were purchased from SBF, Beijing. Mice were raised under specific pathogen-free (SPF) conditions. All animal experiments were approved by the Laboratory Animal Ethics Committee of Guangzhou Medical University. The sepsis model was established by cecal ligation and puncture, as previously described [21, 22]. Briefly, mice were anesthetized with 2% pentobarbital (50 mg/kg intraperitoneal injection). After scraping the abdominal hair with an electric trimmer, the cecum of the mice was identified and exposed and finally ligatured at the middle position. 75% alcohol was used for disinfection and sterilization during surgery. Through-and-through puncture was performed to perforate the cecum with an 18-gauge needle [23]. A small amount of cecal content was extruded into the abdomen. Next, the simple suture method was applied to the abdominal musculature and skin. In the sham group, only the cecum was exposed without ligation or puncture. Finally, colon tissue samples were collected 24 hours after the CLP procedure.

### 2.2. H&E Staining and ELISA Measurement

The colon samples were fixed in 4% paraformaldehyde for 24 h and then processed for sectioning and H&E staining, which were conducted by Servicebio, Guangzhou. Pathological changes were observed under a microscope. The expression levels of IL-1*β*, IL-10, and IL-6 in peritoneal lavage fluid were detected by ELISA (BioLegend, Beijing) [24].

### 2.3. Whole-Transcriptome RNA Sequencing and Bioinformatics Analysis

Total RNAs from the septic mouse colon (*n* = 5) or sham mice (*n* = 5) were extracted, and the RNA quality was strictly controlled. RNA sequencing was performed by Novogene Bioinformatics Technology (Beijing, China). We further predicted the functions of all aberrantly expressed lncRNAs and mRNAs using GO or KEGG analysis. GO is a comprehensive database that describes the gene function, constituent molecular function, biological process, and cellular components. KEGG analysis was aimed at exploring the possible key regulatory pathways for the enrichment of different expressed genes.

### 2.4. Quantitative Real-Time PCR Validation

Total RNA was extracted from colon tissue or cells using the TRIzol reagent (Invitrogen). Reverse transcription was performed using HiScript® Q RT SuperMix with a gDNA wiper (Vazyme), according to the manufacturer's instructions. Real-time PCR was performed using ChamQ SYBR® Color qRT-PCR Master Mix (Q711, Vazyme). The relative expressions of lncRNA were calculated using the 2^-*∆∆*Ct^ method. Primers of lncRNAs were designed and synthesized by Sangon Biotech, China. All primers are shown in Supplementary Table 1. GAPDH was employed as an endogenous control.

### 2.5. Construction of lncRNA-mRNA Coexpression Networks

To validate interactions between the differentially expressed lncRNAs and mRNAs, a lncRNA-mRNA coexpression network was constructed, based on Pearson's correlation coefficient greater than 0.95 and FDR < 0.001. Cystoscope software 3.7.1 was used to construct the coexpression network.

### 2.6. Cell Culture

The MC38 cell line was purchased from iCell Bioscience Inc., China. RAW 264.7 cells were friendly provided by Dr. Min Wu from the University of North Dakota. Cells were cultured in Dulbecco's Minimal Essential Medium (DMEM, Gibco, USA) supplemented with 10% fetal bovine serum (FBS, Gibco, USA), 100U/ml penicillin, and 100 *μ*g/ml streptomycin in a 37°C incubator with 5% CO_2_.

### 2.7. Lipopolysaccharide (LPS) Treatment

RAW 264.7 cells were seeded in a 12-well plate at a density of 2 × 10^5^ and cultured for 24h to 60-70% confluence. Cells were separately pretreated with the ERK inhibitor U0126, NF-*κ*B inhibitor Bay11-7082, or p38 inhibitor SB203580 (purchased from MCE, China) for 30 min and further treated with 200ng/ml LPS (from *Escherichia coli* 0127:B8, purchased from Sigma) for another 24h. After that, cells were collected for qRT-PCR.

### 2.8. Nucleic and Cytoplasmic RNA Extraction

We used the Nucleo-Cytoplasmic Separation Kit (Norgen Biotek Corp., Thorold, ON, Canada) to isolate nucleic and cytoplasmic RNAs following the manufacturer's instructions. The separation effect was identified by detecting cytoplasmic and nucleic markers (*β*-actin and U6), respectively.

### 2.9. Statistical Analysis

Each experiment was repeated at least three times. The significant difference between 2 groups was compared by Student's *t*-test. Statistical analysis was performed using GraphPad Prism 5 software, and *p* < 0.05 was considered statistically significant.

## 3. Result

### 3.1. Colon Injury Validation of the Septic Mouse Model

**Figure 1:**
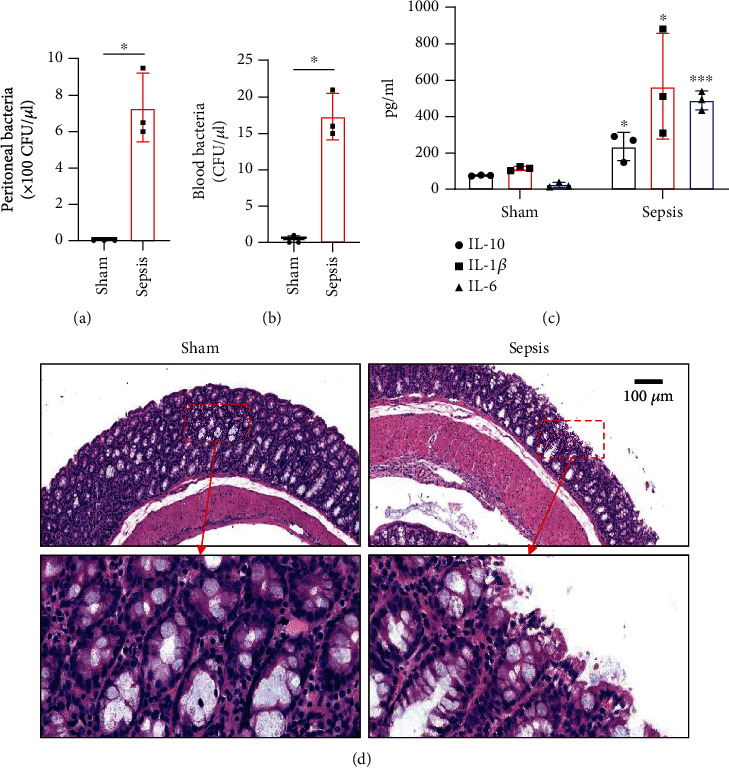
Characteristics of the CLP sepsis mouse model. (a, b) Mice (*n* = 3) were processed for CLP to induce sepsis or sham. 24 h later, bacterial loads in peritoneal lavage and blood were quantified by a colony-forming unit (CFU) assay. (c) Representative images of pathological comparison in colon tissues by H&E staining. Scale bar = 100*μ*m. (d) The expression levels of IL-6, IL-1*β*, and IL-10 of mouse colon homogenates were detected using ELISA. Data are shown as means ± SD. ^∗^*p* < 0.05, ^∗∗∗^*p* < 0.001, Student's *t*-test.

The CLP mouse model is considered the gold standard model in sepsis study [22, 25]. In this study, CLP surgery was performed on a mouse to induce sepsis. After surgery, all mice showed diarrhea, bloody stool, and decreased activity and appetite symptoms (data now shown). Besides, the bacterial loads in the peritoneum and blood were significantly increased in the sepsis group compared with the sham group (Figures 1(a) and 1(b)). H&E staining showed that the integrity of colon tissues was severely destructed in septic mice, as compared with the mice in the sham group (Figure 1(c)). Because CLP sepsis formed through the cecal puncture in the abdominal cavity of mice, the detection of cytokines expressed in the peritoneal fluid of mice was sufficient to prove the occurrence of CLP sepsis [26]. Thus, we examined some inflammatory cytokines, such as IL-10, IL-1*β*, and IL-6, in peritoneal lavage fluid and found that all of the three cytokines were significantly increased in septic mice (Figure 1(d)). These results indicated that our sepsis mouse model was successfully developed.

### 3.2. The Characterization of lncRNA and mRNA in the Septic Colon

**Figure 2:**
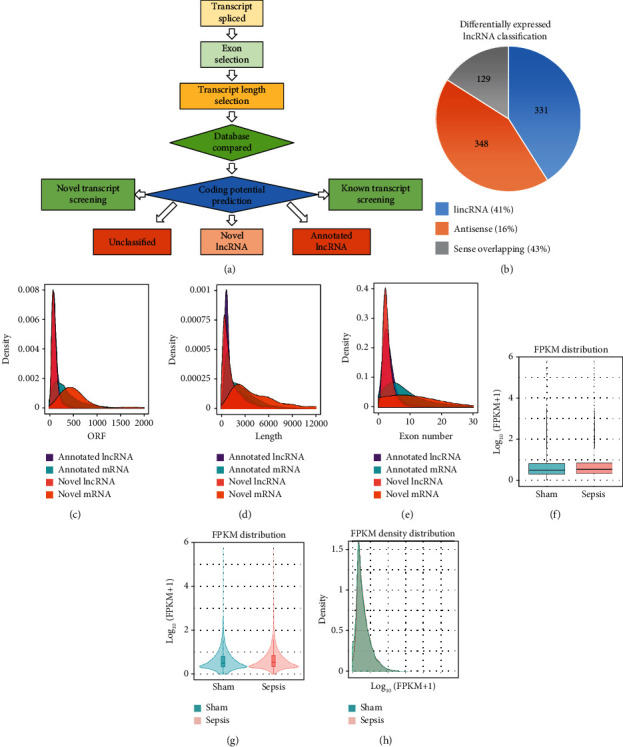
Features of lncRNAs and mRNAs in mouse colon tissue. (a) Flowchart of differentiated lncRNA screening. (b) The classification of differentiated lncRNA between sham and sepsis mice (*n* = 3). (c–e) Density maps of the expression features of (c) opening reading frame (ORF), (d) length, and (e) exon numbers of lncRNA and mRNA in mouse colon tissue. (f) Boxplot, (g) violin plot, and (h) density maps revealing the expression characters of colon tissue from the sepsis group and the sham group. FPKM: fragments per kilobase million.

To identify the expression profile of lncRNA in the mouse septic colon, colon RNA samples (*n* = 5 in each group) from the sepsis group and sham group were sequenced. To optimize the positive rate in identifying lncRNA from the sequenced data, a filtering process (flowchart) was performed to remove transcripts without all the characteristics of lncRNA (Figure 2(a)). And our results revealed that expression levels of 808 lncRNAs are changed in CLP septic mice, including lincRNAs (41%), antisense lncRNAs (16%), and sense overlapping lncRNAs (43%) (Figure 2(b)). Meanwhile, there were 1509 mRNAs identified to be involved in CLP-induced sepsis. Further analysis confirmed that these lncRNAs had a shorter length, fewer exon numbers, and fewer open reading frames (ORF) (Figures 2(c)–2(e)). However, there was no significant difference in transcript levels in the colon of septic or sham mice (Figures 2(f)–2(h)). These results provided a basis for further analysis of differentially expressed lncRNAs and mRNAs upon CLP treatment.

### 3.3. Expression Profile of lncRNA and mRNA

**Figure 3:**
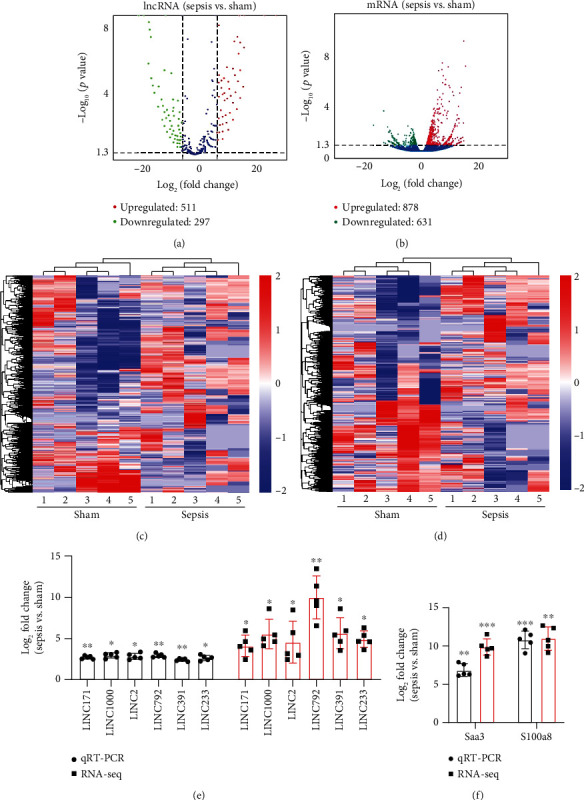
Analysis of differentially expressed lncRNAs and mRNAs. (a, b) Volcano plot showing differentially expressed (*p* < 0.05, ≥|3| fold change) (a) lncRNAs and (b) mRNAs between the sepsis group and sham group. Red indicates the upregulated gene, and green indicates the downregulated gene. (c, d) Hierarchical clustering analysis of (c) lncRNA and (d) mRNA expression profiles between the sepsis group and sham group. Blue represents low expression, and red represents high expression. (e, f) Validation of several differentially expressed lncRNAs and mRNAs by qRT-PCR. The fold change of randomly selected (e) lncRNAs and (f) mRNAs between sepsis and sham mice was detected by qRT-PCR, as compared with RNA sequencing data, respectively. Data are shown as means ± SD. *n* = 5 in each group. ^∗^*p* < 0.05, ^∗∗^*p* < 0.01, Student's *t*-test.

To determine whether lncRNAs were involved in the pathological process of sepsis-induced intestine depression, the expression profiles of lncRNAs and mRNAs were analyzed. We compared the expression level of lncRNAs in the sepsis and sham groups and found that 808 lncRNAs were differently expressed in these two groups, with 511 upregulated lncRNAs and 297 downregulated lncRNAs (Figure 3(a)). Meanwhile, 1509 (878 upregulated and 631 downregulated) differently expressed mRNAs were identified in the sepsis group (Figure 3(b)). The expression profiles of these lncRNAs and mRNAs are shown in a cluster heat map (Figures 3(c) and 3(d)).

In addition, the information of the top 10 upregulated and 10 downregulated lncRNAs are listed in Supplementary Tables 2-3, separately, and the top 20 upregulated and 20 downregulated mRNAs are listed in Supplementary Tables 4-5, separately. These results showed distinct lncRNA and mRNA expression profiles between sepsis and sham mice, implying differences in the pathophysiology of sepsis-induced intestine dysfunction.

### 3.4. Validation of Differentially Expressed lncRNAs by qRT-PCR

To verify the reliability of the sequencing results and provide basis for further study, six differentially expressed upregulated lncRNAs (LINC233, LINC171, LINC2, LINC1000, LINC792, and LINC391) and two upregulated mRNAs (Saa3 and S1008a) were selected and then validated by qRT-PCR. As shown in the results, the expression levels of all these 6 lncRNAs and 2 mRNAs were consistent with the results obtained from sequencing data (Figures 3(e) and 3(f)), which suggested the high quality and validity of RNA sequencing.

### 3.5. Systematic Functional Analysis of Differentially Expressed lncRNAs and mRNAs

**Figure 4:**
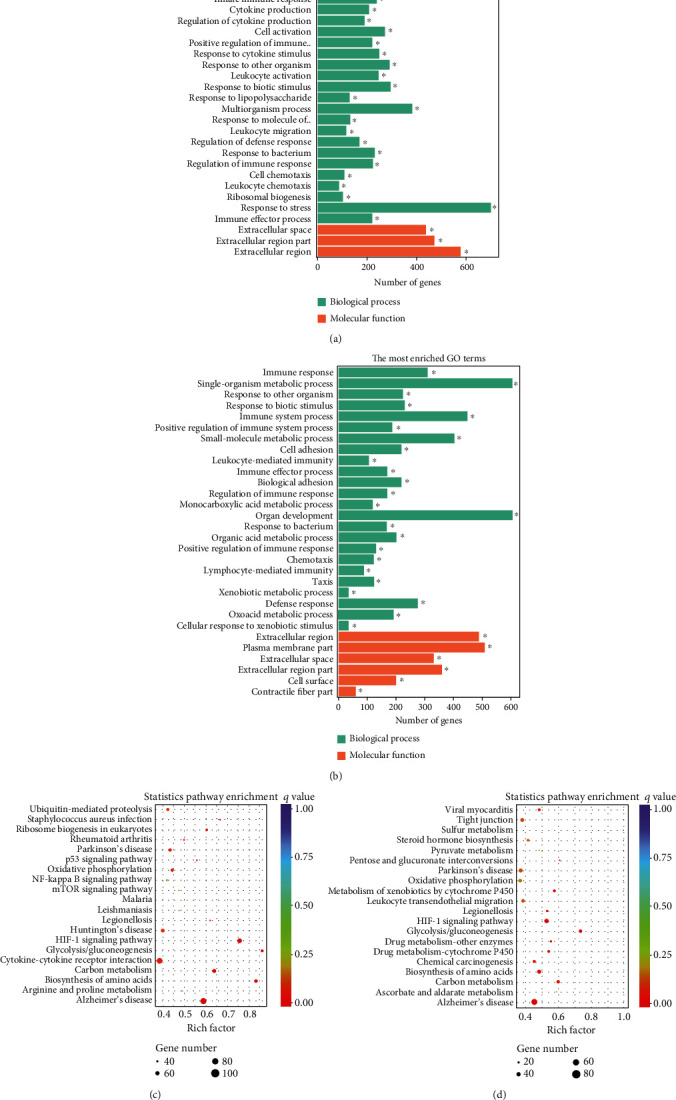
Bioinformatics analysis of differentially expressed lncRNAs and the constructed coexpression network. (a, b) GO enrichment analysis of (a) upregulated lncRNAs and (b) downregulated lncRNAs. Pathway enrichment analysis of (c) upregulated lncRNAs and (d) downregulated lncRNAs.

To elucidate the potential functions of altered lncRNAs and mRNAs in the pathogenesis of sepsis, we performed a Gene Ontology (GO) term enrichment analysis. We classified the GO terms significantly enriched by lncRNAs and mRNAs into three categories: biological process, cellular components, and molecular functions. As shown in Figures 4(a) and 4(b), the top 20 enriched functions of upregulated lncRNAs and downregulated lncRNAs were prominently involved in the immune system process, immune response, etc. To determine whether there were some specific pathways altered in sepsis, we next performed the KEGG enrichment analysis. We found that both upregulated and downregulated lncRNAs have significant correlations with the HIF-1 signaling pathway (Figures 4(c) and 4(d)).

**Figure 5:**
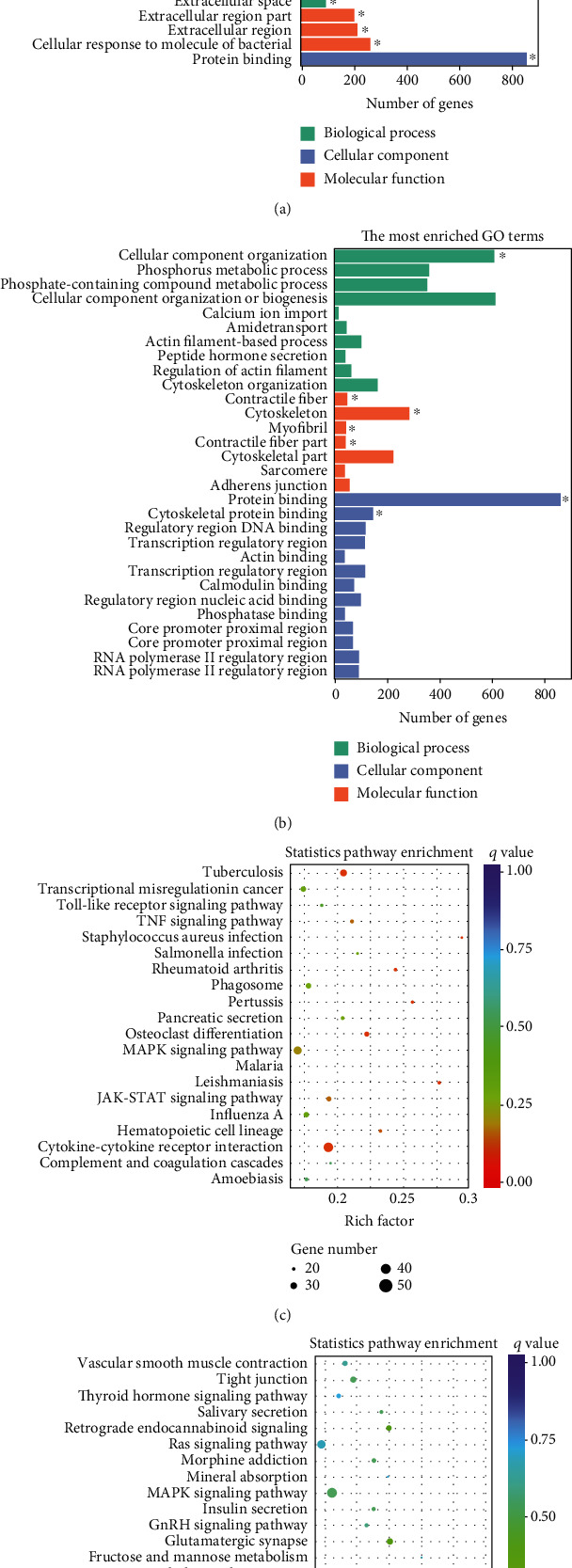
GO enrichment and KEGG pathway analysis of differentially expressed mRNAs in mouse colon tissues. (a) Upexpressed and (b) downexpressed mRNA-enriched biological functions were validated by GO analysis. Key pathways enriched by (c) upexpressed and (d) downexpressed mRNAs were identified by KEGG analysis.

Similarly, upregulated mRNAs are significantly associated with positive regulation of the biological process, positive regulation of the cellular process, etc. (Figure 5(a)). Downregulated mRNAs are related to cellular component organization, cellular component organization or biogenesis, etc. (Figure 5(b)). Interestingly, we found that upregulated lncRNAs and mRNAs have similar significant correlations with the cytokine-cytokine receptor interaction pathway (Figures 5(c) and 5(d) and Supplementary Table 6), while downregulated mRNAs were mainly linked to the MAPK signaling pathway and Ras signaling pathway (Figures 5(c) and 5(d)).

Collectively, the differentially expressed lncRNAs and mRNAs have similar associations with the defense process or immune response upon outside stimulation. These results implied that these inflammatory-related pathways may be involved in intestine dysfunction caused by sepsis.

### 3.6. lncRNA and mRNA Coexpression Networks

lncRNA could form a complex network with target genes, proteins, and other molecules to perform its functions [27, 28]. To visualize the correlation of lncRNAs and mRNAs in the progress of intestinal dysfunction, we constructed the lncRNA-mRNA coexpression network. As shown in Supplementary Figure 1, the coexpression network consisted of 374 nodes and 2495 connections among 28 lncRNAs and 336 mRNAs. The size of the nodes represents the “degree”; the higher the degree, the more significant the molecular function. Large nodes such as TCONS00205497 and TCONS00048204 (LINC233) may play an important role during sepsis progress. In addition, we found that one lncRNA can correlate with multiple mRNAs, and certain mRNA can also be associated with multiple lncRNAs. These results indicate that the pathological process of intestinal dysfunction induced by sepsis is a very complex network of interactions.

### 3.7. The Role of LIN233 in Sepsis-Induced Intestine Depression

**Figure 6:**
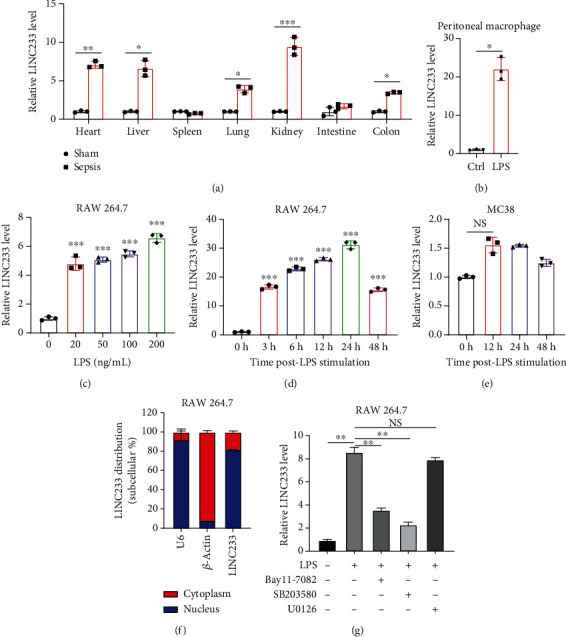
Study of LINC233. (a) Mice (*n* = 3) were executed, and RNAs were extracted from various organs 24h after CLP-induced sepsis or sham. The corresponding LINC233 expression was detected by qRT-PCR. (b) Mouse peritoneal macrophage was isolated from the normal mouse abdomen and then treated with LPS (200ng/ml, 24h); then, LINC233 expression was detected by qRT-PCR. (c, d) RAW 264.7 cells were treated with the indicated dose of LPS for 24h or 200ng/ml LPS for the indicated time. LINC233 expression was detected by qRT-PCR. (e) LINC233 expression level in the LPS- (200ng/ml) treated epithelial cell line MC38. (f) qRT-PCR to detect subcellular expression level of LINC233 in RAW 264.7 cells. *β*-Actin was used as an internal reference of the cytoplasm, and U6 was used as an endogenous control of the nucleus. Expression values are shown as the proportion of cytoplasmic RNA expression vs. nucleic RNA expression. (g) RAW 264.7 cells were pretreated with Bay11-7082 (1*μ*M), SB203580 (5*μ*M), and U0126 (5*μ*M) for 30min, separately. Cells were then treated with LPS (20ng/ml) for another 24h, followed by qRT-PCR analysis of LINC233 expression. Data were shown as means ± SD from triplicates. ^∗^*p* < 0.05, ^∗∗^*p* < 0.01, and ^∗∗∗^*p* < 0.001.

According to the RNA sequencing results, we finally selected the significant upregulated lncRNA (LINC233) to explore the potential immune regulation mechanism. LINC233 was the most significant upregulated lncRNA in our study. And we also found that LINC233 was increased in the heart, liver, lung, kidney, intestine, and colon of the septic mice upon CLP treatment as compared with that in sham mice (Figure 6(a)). At the cellular level, we found that LINC233 was upregulated in both peritoneal macrophages and RAW 264.7 cells (a mouse macrophage cell line) after LPS treatment (Figures 6(b)–6(d)), but not in MC38 cells (epithelial) (Figure 6(e)). These results demonstrated that LINC233 may be involved in the immune function of macrophages, which is of vital importance in host immunity against bacterial infection, to further regulate sepsis progress. The expression site of LINC233 in the RAW 264.7 cells was determined by qRT-PCR to detect subcellular expression level of LINC233 in the cytoplasm and nucleus, and we noticed that LINC233 was mainly localized in the nucleus (Figure 6(f)). To further find out whether LINC233 is involved in influencing the immune function of macrophages, different pathway inhibitors, Bay11-7082, SB203580, or U0126, were used to specifically inhibit NF-*κ*B, p38, or ERK in LPS-treated RAW 264.7 cells. We found that the inhibition of NF-*κ*B or p38 by Bay11-7082 or SB203580 could significantly decrease the expression of LINC233; in contrast, blocking of the ERK pathway by U0126 has little effect on the expression of LINC233 (Figure 6(g)). These results indicate that LINC233 may serve as a potential regulator affecting NF-*κ*B or p38 pathways and may be a clinical target in sepsis treatment.

## 4. Discussion

The molecular mechanisms and gene changes responsible for sepsis-induced colon dysfunction remain largely unknown. We performed whole-transcriptome RNA sequencing in the colon tissues of 5 CLP septic mice and 5 sham mice to analyze differentially expressed lncRNAs and mRNAs. GO analysis of the altered lncRNAs revealed that the immune system process, response to stress, and immune response were mostly enriched. KEGG enrichment analysis results showed that the TNF signaling pathway, JAK-STAT signaling pathway, and NF-*κ*B signaling pathway were mostly enriched in upregulated lncRNAs, while downregulated lncRNAs were involved in cancer, tight junction, antigen processing and presentation, and HIF-1 signaling pathways.

To our knowledge, lncRNAs participate in various diseases, especially in the field of cancer [15]. Currently, lncRNAs whose functional mechanisms have been exactly studied are oncogenic. lncRNAs can act as ceRNA or microRNA sponges to regulate gene expression [29]. For instances, lncRNA MALAT1was first studied as a prognostic marker for lung cancer [30, 31]. H19 not only is a tumor suppressor but also can promote the proliferation and migration of cancer cells [32]. HOTAIR, as an oncogene, is involved in various gastric tumors, driving malignant characteristics of these cancers [33]. In recent years, cancer-related lncRNAs have attracted tremendous attention and extensive researches in the progress of sepsis [34]. For example, HOTAIR promotes TNF-*α* expression and aggravates myocardial damage caused by sepsis [35]. As microRNA sponges, CRNDE regulates the biological function of miR-126-5p, promotes the expression of BCL2L2, and alleviates liver damage induced by sepsis [36]. MALAT1 plays a regulatory role in the inflammatory process of sepsis [37]. H19, SNHG16, and NEAT1 are also involved in sepsis progress [38–40]. The limited number of sepsis-related lncRNAs may impede further study on their complex pathological mechanism. Sequencing technology, as an advanced approach, can decipher the different expression profiles of various diseases. Until now, transcriptome analyses of the septic myocardium in rats, septic kidney of patients, sepsis encephalopathy of rats, and septic intestine of mice have been completed [41–43]. It is suggested that differential expression of lncRNAs was associated with organ damage induced by sepsis. The colon is considered to be a driving factor of multiple-organ damage in sepsis, resulting in a high mortality. Previous researches of lncRNAs have provided a new perspective for the treatment of intestinal injury caused by sepsis. Sun et al. found that H19 lncRNA can promote mucosal regeneration [42]. Su et al. revealed that H19 lncRNA promotes the recovery of intestinal barrier function by regulating the expression of AQP3 [44]. However, the lncRNA profile in sepsis-induced colon depression has never been sequenced. In order to fill this gap, our study analyzed the features of lncRNA and mRNA in sepsis-induced colon depression. To perfectly replicate the pathological process of a septic patient, we choose the CLP sepsis mouse model, and we observed a significant increase in inflammatory cytokine levels and destruction of the colon, which is consistent with previous reports.

We collected tissue samples 24 hours after the establishment of the CLP-induced sepsis mouse model and then performed RNA sequencing. The standard analysis process of the RNA-seq mainly includes quality control, comparison, splicing, screening, quantitative analysis, analysis of the significance of difference, functional enrichment, and other aspects. Finally, we identified 808 altered lncRNAs (511 upregulated and 297 downregulated) and 1509 mRNAs (878 upregulated and 631 downregulated) in the CLP sepsis mouse colon in comparison to the sham group mouse colon through RNA-seq. GO analysis of altered mRNAs reveals that the upregulated mRNA enriched terms were mostly occupied by defense response, inflammatory response, and multiorganism process. Downregulated mRNAs were mainly enriched in cellular component organization, cytoskeleton, and protein binding. The underlying function of lncRNAs was poorly understood. KEGG enrichment results showed that the TNF signaling pathway and JAK-STAT pathway were enriched by upregulated lncRNAs and upregulated mRNAs, which implied that these differentially expressed lncRNAs may have a potential association with these upregulated mRNAs. At the same time, we found that the upregulated lncRNAs and mRNA target genes had a similar significant correlation with the cytokine-cytokine receptor interaction pathway. These signaling pathways have been broadly studied during sepsis progress [45–47]. It was indicated that lncRNAs may participate in intestine dysfunction by regulating these signaling pathways.

The qRT-PCR experiment validated the high reliability of our sequencing results. After that, we focused on one specific lncRNA, LINC233, which was the most upregulated lncRNA, which was located at chr13:55184395-55189175. By filtering the target genes located near the LINC233, we focused on UIMC1 (ubiquitin interaction motif containing 1). RAP80/UIMC1 is a DNA damage repair (DDR) factor related to cell biological processes, such as cell proliferation and apoptosis [48]. However, the role of UIMC1 in sepsis remains unclear. Moreover, the coexpressed target genes of LINC233 include CD47, Lcn2, and Sphk1. Previous studies have confirmed that CD47, Lcn2, and Sphk1 were involved in the sepsis process [49–51]. For target genes, interactions between differentially expressed mRNAs and lncRNAs were predicted and merged with the target genes. Coexpression of lncRNAs and mRNAs was selected to construct mRNA-lncRNA interaction networks [52]. CD47 was decreased in platelets of septic mice which on the other hand can increase the mortality of mice suffering sepsis [53]. Lcn2 can combat gut-origin sepsis via maintaining homeostasis of the microbiota to alleviate gut barrier injury [54]. Therefore, we speculated that LINC233 might participate in CLP-induced sepsis by interacting with this protein-coding gene. The functions of lncRNAs were associated with their subcellular localizations [55]. Nucleic and cytoplasmic RNA extraction and qRT-PCR experiment validated that LINC233 was a nuclearly localized lncRNA. Nuclear-retained lncRNAs were shown to play a role in chromatin and nuclear structure organization, transcription, and posttranscription modification, which provide guidance for the study of the mechanism of LINC233 [56].

There are some limitations in our study: First, only five samples from sepsis and sham groups were subjected to RNA sequencing. In order to improve the reliability and validity of sequencing, more samples are greatly needed. Second, due to poor conservation of lncRNAs among species, our sequencing data need to be evaluated more rigorously. Third, to investigate the function of LINC233 in depth, future experiments of knockdown or overexpression are needed. Considering the nuclear localization characteristics of LINC233, the interference assay should be specifically designed for nuclear lncRNAs.

To our knowledge, our work appears to be the first study to use RNA sequencing to discuss the differentially expressed profiles of lncRNAs and mRNAs in colon tissue of a CLP-induced sepsis mouse. Our research may provide some new diagnostic and therapeutic targets for sepsis-induced colon damage or other colitis-related phenotypes. Therefore, numerous works will be needed to study the interaction of these novel lncRNAs with mRNAs to reveal the exact functions and molecular mechanisms in the future.

## Data Availability

The data used to support the findings of this study are available from the corresponding author upon request.

## Supplementary Materials

Supplementary Materials Supplementary Table 1: primers used for qRT-PCR of lncRNAs and mRNAs. Supplementary Table 2: information of the top 10 upregulated lncRNAs. Supplementary Table 3: information of the top 10 downregulated lncRNAs. Supplementary Table 4: information of the top 20 upregulated mRNAs. Supplementary Table 5: information of the top 20 downregulated mRNAs. Supplementary Table 6: top 30 pathway enrichment of upregulated lncRNAs. Supplementary Figure 1: lncRNA-mRNA coexpression network.
