# Supplementary material for: Expression Profiling of Long Noncoding RNA and Messenger RNA in a Cecal Ligation and Puncture-Induced Colon Injury Mouse Model
Source: Mediators Inflamm. 2020 Nov 3;2020:8925973. doi: 10.1155/2020/8925973 (PMC7657679; doi:10.1155/2020/8925973)
Supplement: Supplementary Materials — Supplementary Table 1: primers used for qRT-PCR of lncRNAs and mRNAs. Supplementary Table 2: information of the top 10 upregulated lncRNAs. Supplementary Table 3: information of the top 10 downregulated lncRNAs. Supplementary Table 4: information of the top 20 upregulated mRNAs. Supplementary Table 5: information of the top 20 downregulated mRNAs. Supplementary Table 6: top 30 pathway enrichment of upregulated lncRNAs. Supplementary Figure 1: lncRNA-mRNA coexpression network. [file 8925973.f1.docx]

**Supplementary Table 1: Primers Used for qRT-PCR of LncRNAs and mRNAs**

| **LncRNAs** | **Primer** |
| --- | --- |
| LINC171-Forward | CTGTCCATCACTGCACTGCTGTC |
| LINC171-Reverse | CCGATGGCTGACTAACGCTCTTG |
| LINC1000-Forward | TCAACCTCCAGGCTACTCTCATCC |
| LINC1000-Reverse | TGCACATAGTGGCTTCTGGTCAAG |
| LINC2-Forward | TTCATACTCCTCCACCGACCAGTG |
| LINC2-Reverse | CGTCCTTGACCAGAAGCCACTATG |
| LINC391-Forward | CCGATGGCTGACTAACGCTCTTG |
| LINC391-Reverse | CGCCTTCCGCTCACCTTCAAC |
| LINC792-Forward | CGTCCTTGACCAGAAGCCACTATG |
| LINC792-Reverse | GCCTTGGATGCTCTTGAGACTGC |
| LINC233-Forward | ACCAATGGCTGACTAACGCTCTTG |
| LINC233-Reverse | CTGTCCATCACTGCACTGCTGTC |
| Mus S100a8 -Forward | AAATCACCATGCCCTCTACAAG |
| Mus S100a8 -Reverse | CCCACTTTTATCACCATCGCAA |
| Mus Saa3 -Forward | TGCCATCATTCTTTGCATCTTGA |
| Mus Saa3-Reverse | CCGTGAACTTCTGAACAGCCT |
| Mus-GAPDH-Forward | AACTTTGGCATTGTGGAAGG |
| Mus-GAPDH-Reverse | CACATTGGGGGTAGGAACAC |

**Supplementary Table 2: Information of the Top 10 Up-Regulated LncRNAs**

| **Transcript ID** | **Transcript Name** | **Gene Name** | **Transcript Locus** | **VPV FPKM** | **VPC FPKM** | **Log_2_ Fold Change** | **P Value** |
| --- | --- | --- | --- | --- | --- | --- | --- |
| TCONS_00048204 | LINC233 | XLOC_024919 | 13:55184395-55189175 | 182.4588363 | 0 | 18.51256967 | 3.50E-11 |
| TCONS_00048203 | LINC232 | XLOC_024919 | 13:55184313-55189175 | 48.22765667 | 2.008556667 | 4.608017881 | 0.024941505 |
| TCONS_00104950 | LINC464 | XLOC_052511 | 2:75638376-75646942 | 75.517915 | 0 | 18.06236135 | 3.83E-10 |
| ENSMUST00000198156 | S100a8-202 | S100a8 | 3:90669311-90669964 | 71.93456267 | 0.25521 | 7.716105183 | 0.000105938 |
| ENSMUST00000136509 | Lcn2-202 | Lcn2 | 2:32386556-32387759 | 59.16579067 | 0.242003 | 7.899059268 | 6.45E-05 |
| ENSMUST00000144569 | Lcn2-203 | Lcn2 | 2:32384639-32385999 | 47.44600733 | 0.106477333 | 8.330598458 | 3.78E-05 |
| TCONS_00034621 | LINC171 | XLOC_017606 | 11:86811751-86816395 | 23.62828633 | 1.064026 | 4.049832946 | 0.012230012 |
| TCONS_00104952 | LINC465 | XLOC_052511 | 2:75638379-75646942 | 13.84597633 | 0 | 15.61598426 | 7.07E-08 |
| ENSMUST00000176184 | Rpl41-202 | Rpl41 | 10:128548121-128549159 | 7.791827 | 0 | 11.95194021 | 0.000166674 |
| TCONS_00104951 | LINC463 | XLOC_052511 | 2:75638376-75646942 | 6.656219 | 0 | 14.53754267 | 9.97E-05 |

**Supplementary Table 3: Information of the Top 10 Down-Regulated LncRNAs**

| **Transcript ID** | **Transcript Name** | **Gene Name** | **Transcript Locus** | **VPV FPKM** | **VPC FPKM** | **Log_2_ Fold Change** | **P Value** |
| --- | --- | --- | --- | --- | --- | --- | --- |
| TCONS_00126554 | Riiad1-OT2 | Riiad1 | 3:94464983-94480267 | 0 | 3.642194 | -14.52493313 | 9.16E-05 |
| ENSMUST00000182859 | Kat6b-208 | Kat6b | 14:21499854-21518643 | 0 | 1.234802 | -13.67496725 | 4.98E-06 |
| ENSMUST00000181142 | 9030616G12Rik-201 | 9030616G12Rik | 10:128971197-128978115 | 0 | 0.793369333 | -11.2827516 | 0.000167451 |
| TCONS_00147546 | Tnrc18-AS5 | Tnrc18 | 5:142812144-142817584 | 0 | 0.721606 | -11.09545867 | 0.000161999 |
| ENSMUST00000182058 | Dclre1a-202 | Dclre1a | 19:56529169-56547155 | 0.001107667 | 0.683420667 | -9.086350711 | 0.000157866 |
| ENSMUST00000183066 | Dleu2-211 | Dleu2 | 14:61603472-61682302 | 0 | 0.469024667 | -14.06338162 | 0.000288168 |
| TCONS_00119404 | LINC527 | XLOC_059365 | 3:52649901-52689121 | 0 | 0.452511333 | -13.35695488 | 8.86E-06 |
| ENSMUST00000212465 | Gm32352-201 | Gm32352 | 8:119659475-119670881 | 0 | 0.415342333 | -11.09828978 | 0.000169302 |
| ENSMUST00000128197 | Gria3-205 | Gria3 | X:41400854-41613099 | 0 | 0.37491 | -15.03236914 | 4.99E-05 |
| TCONS_00208098 | LINC979 | XLOC_103012 | X:78967167-79001124 | 0 | 0.372120333 | -12.8873622 | 2.67E-05 |

**Supplementary Table 4: Information of the Top 20 Up-Regulated mRNAs**

| **Transcript ID** | **Transcript Name** | **Gene Name** | **Transcript Locus** | **VPV FPKM** | **VPC FPKM** | **Log_2_ Fold Change** | **P Value** |
| --- | --- | --- | --- | --- | --- | --- | --- |
| ENSMUST00000006956 | Saa3-201 | Saa3 | 7:46711998-46715700 | 9284.90918 | 12.33342233 | 9.205806968 | 2.25E-07 |
| ENSMUST00000034215 | Mt1-201 | Mt1 | 8:94179082-94180327 | 2853.823852 | 31.665453 | 6.118687698 | 5.10E-05 |
| ENSMUST00000034214 | Mt2-201 | Mt2 | 8:94172664-94173568 | 1783.923726 | 13.46514367 | 7.005557617 | 8.54E-06 |
| ENSMUST00000069927 | S100a8-201 | S100a8 | 3:90668978-90670035 | 1717.801056 | 1.002921 | 10.46126853 | 2.03E-06 |
| ENSMUST00000069960 | S100a9-201 | S100a9 | 3:90692632-90695711 | 1621.29541 | 1.426162 | 9.962797969 | 5.30E-08 |
| ENSMUST00000050785 | Lcn2-201 | Lcn2 | 2:32384633-32387797 | 1252.393331 | 4.050471333 | 8.267585411 | 1.79E-06 |
| ENSMUST00000021506 | Serpina3n-201 | Serpina3n | 12:104406729-104414329 | 439.706828 | 5.084313 | 6.399697683 | 2.19E-05 |
| ENSMUST00000115342 | Timp1-202 | Timp1 | X:20870166-20874733 | 261.4116567 | 1.451428667 | 7.410226899 | 4.37E-06 |
| ENSMUST00000009530 | Timp1-201 | Timp1 | X:20870221-20874735 | 229.7475637 | 0.666728667 | 8.516771677 | 8.04E-05 |
| ENSMUST00000031318 | Cxcl5-201 | Cxcl5 | 5:90759378-90761624 | 215.879869 | 0.351059667 | 8.611158599 | 3.56E-06 |
| ENSMUST00000070832 | Wfdc21-201 | Wfdc21 | 11:83746940-83752642 | 128.498109 | 1.783876 | 6.114071836 | 0.000213303 |
| ENSMUST00000031326 | Cxcl3-201 | Cxcl3 | 5:90786103-90788093 | 125.3428923 | 0.037732 | 11.1175737 | 1.21E-06 |
| ENSMUST00000117167 | S100a9-202 | S100a9 | 3:90692632-90695721 | 118.9337247 | 0 | 17.69145103 | 2.81E-10 |
| ENSMUST00000020161 | Arg1-201 | Arg1 | 10:24915221-24927484 | 112.6039897 | 0.283581333 | 8.894803652 | 8.20E-07 |
| ENSMUST00000065666 | Retnlg-201 | Retnlg | 16:48872621-48874496 | 112.218511 | 0.631788 | 7.545092986 | 2.38E-05 |
| ENSMUST00000029421 | Ptx3-201 | Ptx3 | 3:66219910-66225805 | 108.0322573 | 0.158091 | 9.410578242 | 2.58E-05 |
| ENSMUST00000063062 | Chil3-201 | Chil3 | 3:106147554-106167564 | 84.98689 | 0.262433 | 8.090982582 | 1.80E-06 |
| ENSMUST00000198156 | S100a8-202 | S100a8 | 3:90669311-90669964 | 71.93456267 | 0.25521 | 7.716105183 | 0.000105938 |
| ENSMUST00000028881 | Il1b-201 | Il1b | 2:129364570-129371139 | 71.84666567 | 0.809356667 | 6.208419901 | 0.000101553 |
| ENSMUST00000102845 | Rps27a-202 | Rps27a | 11:29545846-29548109 | 67.37149033 | 0.223341333 | 8.245603766 | 0.000137729 |

**Supplementary Table 5: Information of the Top 20 Down-Regulated mRNAs**

| **Transcript ID** | **Transcript Name** | **Gene Name** | **Transcript Locus** | **VPV FPKM** | **VPC FPKM** | **Log_2_ Fold Change** | **P Value** |
| --- | --- | --- | --- | --- | --- | --- | --- |
| ENSMUST00000217820 | Arhgap5-202 | Arhgap5 | 12:52504342-52568835 | 0 | 9.519309 | -18.33675328 | 6.47E-12 |
| ENSMUST00000113795 | Igsf5-206 | Igsf5 | 16:96361794-96525580 | 0 | 4.225362 | -18.1643013 | 3.67E-06 |
| ENSMUST00000108821 | 2210407C18Rik-202 | 2210407C18Rik | 11:58608204-58616075 | 0 | 37.702095 | -17.81804566 | 8.17E-06 |
| ENSMUST00000116457 | Cd82-205 | Cd82 | 2:93419116-93462946 | 0 | 12.382468 | -17.810047 | 4.68E-06 |
| ENSMUST00000043111 | Edil3-201 | Edil3 | 13:88821642-89323223 | 0 | 0.928711 | -17.58653161 | 5.48E-06 |
| ENSMUST00000213124 | Higd1a-202 | Higd1a | 9:121849539-121857535 | 0 | 30.33112933 | -17.18807563 | 3.05E-10 |
| ENSMUST00000226119 | Ctnnd2-203 | Ctnnd2 | 15:30173051-31028041 | 0 | 0.353423333 | -16.9600464 | 9.15E-06 |
| ENSMUST00000003741 | Rps6ka1-201 | Rps6ka1 | 4:133847294-133887784 | 0 | 4.853723 | -16.92843532 | 6.66E-10 |
| ENSMUST00000090314 | Dgki-202 | Dgki | 6:36846981-37300184 | 0 | 0.620016667 | -16.84675893 | 1.23E-05 |
| ENSMUST00000160859 | Ocln-205 | Ocln | 13:100498114-100552481 | 0 | 3.190214667 | -16.80988592 | 1.28E-09 |
| ENSMUST00000138760 | Tenm4-209 | Tenm4 | 7:96210154-96553482 | 0 | 0.714537667 | -16.6578142 | 1.21E-05 |
| ENSMUST00000121404 | Adtrp-202 | Adtrp | 13:41764133-41847684 | 0 | 2.360089667 | -16.47707117 | 1.63E-07 |
| ENSMUST00000013667 | Bcas1-201 | Bcas1 | 2:170347148-170427828 | 0 | 2.524803333 | -16.41677569 | 5.49E-06 |
| ENSMUST00000097547 | Odr4-204 | Odr4 | 1:150361313-150392762 | 0 | 6.326883667 | -16.36029581 | 1.49E-05 |
| ENSMUST00000090925 | Baz2b-201 | Baz2b | 2:59899445-60006187 | 0 | 1.696090667 | -16.227858 | 1.73E-05 |
| ENSMUST00000107196 | Dlg2-204 | Dlg2 | 7:91090706-92449247 | 0 | 0.071874667 | -16.10660128 | 3.20E-08 |
| ENSMUST00000140159 | Oasl1-203 | Oasl1 | 5:114923435-114936067 | 0 | 6.866428 | -16.08163996 | 3.30E-05 |
| ENSMUST00000160910 | Zeb1-205 | Zeb1 | 18:5593602-5759057 | 0 | 0.966545333 | -16.03994565 | 2.07E-05 |
| ENSMUST00000112673 | Pip5k1b-202 | Pip5k1b | 19:24294794-24555842 | 0 | 0.379664 | -16.02920627 | 2.89E-05 |
| ENSMUST00000111098 | Wt1-201 | Wt1 | 2:105130883-105173612 | 0 | 3.456022 | -15.94725926 | 3.62E-05 |

**Supplementary Table 6: Top 30 Pathway Enrichment of Up-Regulated LncRNAs**

| **Term** | **Database** | **ID** | **Input Number** | **Background Number** | **P Value** |
| --- | --- | --- | --- | --- | --- |
| HIF-1 signaling pathway | KEGG PATHWAY | mmu04066 | 84 | 111 | 1.06E-11 |
| Biosynthesis of amino acids | KEGG PATHWAY | mmu01230 | 65 | 78 | 1.41E-10 |
| Glycolysis / Gluconeogenesis | KEGG PATHWAY | mmu00010 | 56 | 65 | 1.12E-09 |
| Alzheimer's disease | KEGG PATHWAY | mmu05010 | 102 | 173 | 2.34E-09 |
| Carbon metabolism | KEGG PATHWAY | mmu01200 | 71 | 111 | 5.95E-08 |
| Ribosome biogenesis in eukaryotes | KEGG PATHWAY | mmu03008 | 49 | 81 | 1.86E-05 |
| Staphylococcus aureus infection | KEGG PATHWAY | mmu05150 | 34 | 51 | 8.62E-05 |
| Legionellosis | KEGG PATHWAY | mmu05134 | 36 | 58 | 0.000155 |
| p53 signaling pathway | KEGG PATHWAY | mmu04115 | 38 | 68 | 0.000493 |
| Oxidative phosphorylation | KEGG PATHWAY | mmu00190 | 60 | 135 | 0.001552 |
| Rheumatoid arthritis | KEGG PATHWAY | mmu05323 | 41 | 82 | 0.001589 |
| Parkinson's disease | KEGG PATHWAY | mmu05012 | 64 | 147 | 0.001648 |
| Cytokine-cytokine receptor interaction | KEGG PATHWAY | mmu04060 | 102 | 264 | 0.002263 |
| Ubiquitin mediated proteolysis | KEGG PATHWAY | mmu04120 | 59 | 139 | 0.00377 |
| Huntington's disease | KEGG PATHWAY | mmu05016 | 73 | 182 | 0.004323 |
| Arginine and proline metabolism | KEGG PATHWAY | mmu00330 | 29 | 59 | 0.008484 |
| Leishmaniasis | KEGG PATHWAY | mmu05140 | 31 | 65 | 0.00919 |
| mTOR signaling pathway | KEGG PATHWAY | mmu04150 | 29 | 61 | 0.011846 |
| Malaria | KEGG PATHWAY | mmu05144 | 24 | 48 | 0.013529 |
| NF-kappa B signaling pathway | KEGG PATHWAY | mmu04064 | 42 | 100 | 0.014664 |
| African trypanosomiasis | KEGG PATHWAY | mmu05143 | 18 | 34 | 0.020316 |
| Glycine, serine and threonine metabolism | KEGG PATHWAY | mmu00260 | 20 | 40 | 0.022849 |
| NOD-like receptor signaling pathway | KEGG PATHWAY | mmu04621 | 26 | 58 | 0.027561 |
| Metabolic pathways | KEGG PATHWAY | mmu01100 | 384 | 1256 | 0.029444 |
| Phenylalanine metabolism | KEGG PATHWAY | mmu00360 | 13 | 23 | 0.031904 |
| Proteasome | KEGG PATHWAY | mmu03050 | 21 | 45 | 0.033392 |
| Cysteine and methionine metabolism | KEGG PATHWAY | mmu00270 | 19 | 40 | 0.037009 |
| RNA degradation | KEGG PATHWAY | mmu03018 | 32 | 78 | 0.037469 |
| TNF signaling pathway | KEGG PATHWAY | mmu04668 | 42 | 109 | 0.039476 |


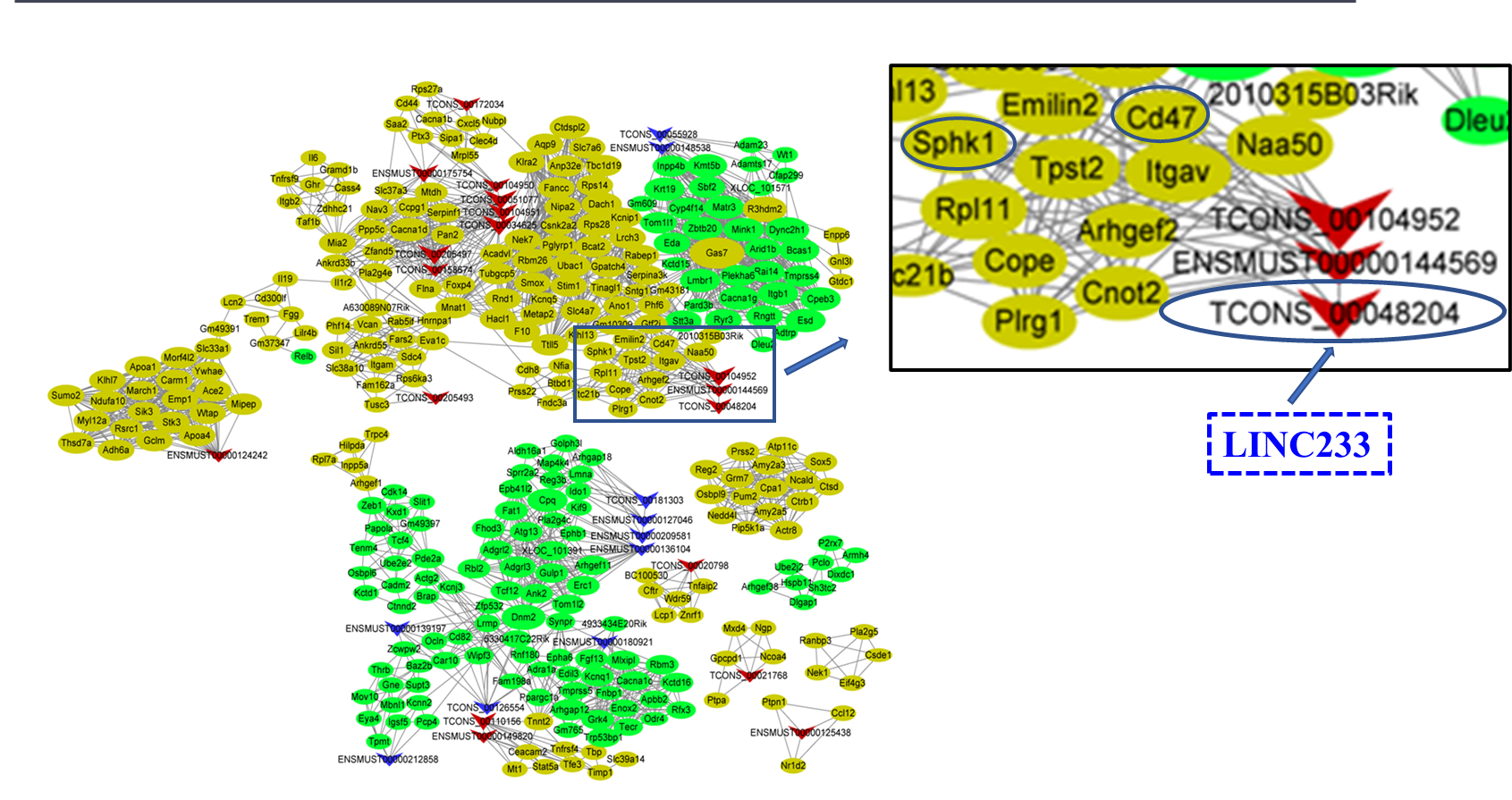


**Supplementary Fig. 1.** LncRNA-mRNA co-expression network. 28 LncRNAs and 336 mRNAs were included in the network (|COR|>0.95; q<0.001). The red arrow represents up-regulated LncRNAs and the blue arrow represents down-regulated LncRNAs, yellow elliptic represents up-regulated mRNAs, green elliptic represents down-regulated mRNAs. The size of the nodes represents the “degree”, larger node shows more co-expressed genes or LncRNAs.
